# Fungal holobionts as blueprints for synthetic endosymbiotic systems

**DOI:** 10.1371/journal.pbio.3002587

**Published:** 2024-04-12

**Authors:** Laila P. Partida-Martínez

**Affiliations:** Centro de Investigación y de Estudios Avanzados (Cinvestav)–Irapuato, Irapuato, Mexico; University of Texas Austin, UNITED STATES

## Abstract

Rhizopus microsporus is an example of a fungal holobiont, harboring bacterial and viral endosymbionts. This Perspective article discusses how these microbial allies increase pathogenicity and defense and control asexual and sexual reproduction in the fungus.

## Teaser

*Rhizopus microsporus* is an example of a fungal holobiont. Strains of this species can harbor bacterial and viral endosymbionts inherited by the next generation. These microbial allies increase pathogenicity and defense and control asexual and sexual reproduction.

Fungi are highly diverse and carry out many critical tasks within the ecosystem, from the decomposition of organic matter to the translocation of nutrients through their hyphae and the connection of distant niches in the soil. However, fungi do not live in isolation; instead, they form close associations with plants and animals as part of their complex microbiota. Fungi are well known for their role as essential mycorrhizal symbionts to most vascular plants and for their lichen symbioses with algae or cyanobacteria; what is less well known is their symbiotic relationships with microbes, both bacteria and RNA viruses [1,2]. Bacterial endosymbionts in fungi were first observed via microscopy in 1970 [3], and more recent discoveries have revealed that these endosymbiotic bacteria can underlie prominent traits in certain fungi [1,4]. By contrast, most mycoviruses, first formally described in 1962 [5], do not have marked effects on their host (although some can reduce fungal growth and virulence).

**Fig 1:**
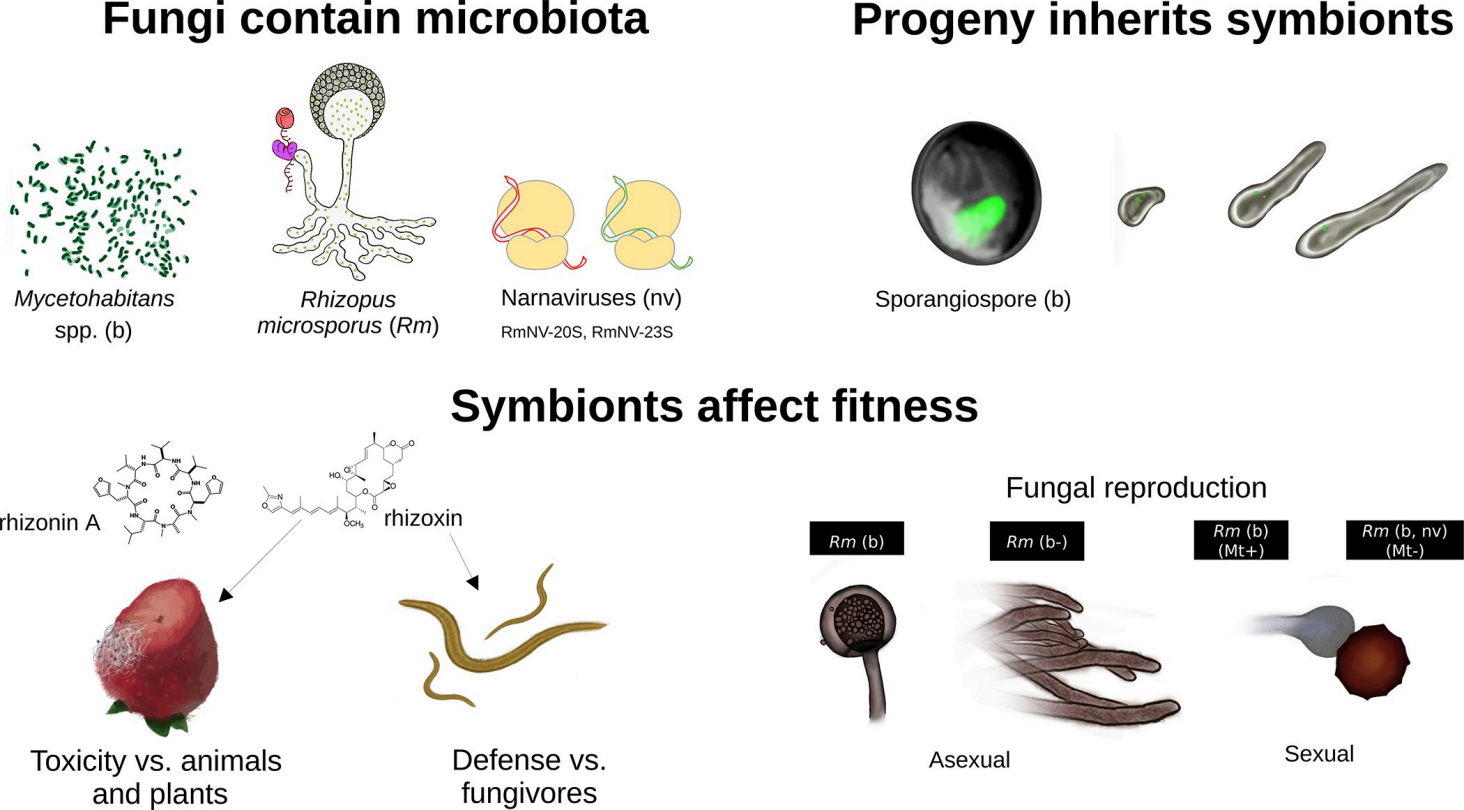
The holobiont *Rhizopus microsporus*. Fungi contain microbiota: *R*. *microsporus* (*Rm*) can live in symbiosis with *Mycetohabitans* bacteria and the RmNV-20S and RmNV-23S narnaviruses. **Progeny inherits symbionts**: A bacterial cell is enclosed inside the sporangiospore. The sporangiospore germinates, and 2 bacterial cells are visible in the growing hypha. **Symbionts affect fitness**: The toxins rhizonin and rhizoxin, produced by *Mycetohabitans*, increase *R*. *microsporus*’s pathogenicity towards animals and plants, while rhizoxin also serves as a defense against fungivores. *Mycetohabitans* control asexual fungal reproduction. No sporangia or sporangiospores are formed without symbiotic bacteria (as shown in the drawing labeled *Rm* (b-)). Sexual zygospores are successfully produced when *Mycetohabitans* and narnaviruses are present. The strain *Rm* (Mt+) harbors *Mycetohabitans* (b), while the strain *Rm* (Mt-) harbors *Mycetohabitans* and the 2 narnaviruses (b, nv).

*Rhizopus microsporus* is a well-studied example of a fungus that can harbor bacterial and viral endosymbionts, known as a fungal holobiont (Fig 1). *Rhizopus* species are used to produce fermented foods, enzymes, and metabolites. Still, they can also be pathogenic for crops (including strawberries, sweet potatoes, and rice) and cause fatal infections in immunocompromised humans. Among their notable traits is the capacity to produce mycotoxins, including rhizoxins, rhizonins, and their derivatives.

Interestingly, research on rhizoxin-producing and non-producing strains of *Rhizopus* revealed that the biosynthetic genes involved in rhizoxin production are not of fungal origin. Instead, all rhizoxin-producing strains are colonized by bacterial symbionts that harbor bacterial polyketide biosynthetic genes capable of producing rhizoxins [6]. Another striking discovery is that strains of *R*. *microsporus* that lack their bacterial symbionts can no longer reproduce asexually and form sporangia and sporangiospores [7]. Sporulation resumes only with the reestablishment of the fungus–bacteria symbiosis [7]. Indeed, bacterial symbionts are inherited in the sporangiospores (Fig 1), ensuring their transmission to the following generations [7].

Sexual reproduction in *R*. *microsporus* requires the contact of 2 compatible partners (a mating type positive (Mt+) and a mating type negative (Mt-) strain) and the collaborative production of trisporic acid, a sex hormone, for the formation of the zygospores (Fig 1). Remarkably, the tester strains for sexual reproduction in *R*. *microsporus*, as proposed by Schipper and Stalpers in 1984 [8], are symbiotic. The lack of bacterial symbionts in both sexual partners (Mt+) and (Mt-) drastically affects the production of zygospores [9], suggesting that symbiotic bacteria influence sexual success in this species.

These endofungal bacteria were first taxonomically classified as members of *Burkholderia* and now form the novel genus *Mycetohabitans* within *Burkholderia* sensu lato [10]. In natural environments, members of *Mycetohabitans* had only been found in association with several strains of *R*. *microsporus* and with one strain of *R*. *delemar* (subphylum Mucoromycotina) [11]. Recently, however, *Mycetohabitans* spp. have been reported in association with strains of *Mortierella verticillata* from the sister subphylum Mortierellomycotina [12], suggesting that these symbionts might be more widely distributed in diverse lineages of the early-diverging phylum Mucoromycota. Importantly, these newly discovered fungus–bacteria symbioses might be expected to produce distinct phenotypes, given that in *R*. *delemar*, *Mycetohabitans* does not produce rhizoxin and asexual reproduction is not dependent on symbiotic bacteria [11].

*R*. *microsporus* can also harbor viral symbionts: *R*. *microsporus* 20S *narnavirus* and *R*. *microsporus* 23S *narnavirus* [13]. These narnaviruses belong to the simplest type of known RNA viruses. Each viral genome consists of a positive single-strand of RNA that codes for a single protein, an RNA-dependent RNA polymerase that drives viral replication. These narnaviruses are highly transcribed during fungal development, especially when a rich substrate is available. The fungal progeny also vertically inherit narnaviruses via the sporangiospores and zygospores [13]. In *R*. *microsporus*, these narnaviruses reduce the number of asexual sporangiospores produced, as they impose a metabolic cost to the fungus [13,14]. Notably, sexual reproduction is also clearly influenced by the presence of *Mycetohabitans* and the narnaviruses. Zygospore production is compromised if both bacterial and viral symbionts are eliminated in the partner (Mt-) [13], similar to the results obtained when both sexual partners lack *Mycetohabitans* [9]. However, exactly how bacteria and viruses participate in the sexual reproduction of *Rhizopus* is unclear. As far as I know, there is no successful sexual mating pair in *R*. *microsporus*, where both partners are naturally asymbiotic. Thus, further investigations are needed to reveal how these fungi might manage to have sex without symbionts.

The fact that not all fungi and *Rhizopus* harbor endosymbionts prompts several questions. What is the distribution and frequency of bacterial and viral symbionts in natural fungal populations? Is viral symbiosis more rare than symbiosis with bacteria? Does establishing one of these bacterial or viral symbioses affect the other one? Which one arose first? To what extent have each of these symbioses contributed to the diversification of fungi and *Rhizopus*? Can these bacterial and viral symbionts (or some of their genes) be transmitted horizontally to other fungi or organisms like plants, insects, and nematodes? Have some symbiotic genes moved to the nuclear fungal genome (as has happened with mitochondrial genes)? What drives symbiotic genome evolution in fungal hosts? How do symbiotic and asymbiotic fungi interact in populations? How do they influence other organisms they interact with?

All these exciting questions require that we continue our search for more of these symbioses in natural ecosystems to gain a broader view of the patterns that govern symbioses’ establishment and endurance. Only a few population-level studies exist and only for certain fungal groups. Furthermore, few studies simultaneously address the holobiont nature of fungi by screening for both bacterial and viral endosymbionts. To move the field forward, we need to combine genomics, transcriptomics, and metabolomics to read the dialogues occurring in these microbial assemblies in depth. Also, visualization and single-cell technologies, microfluidics, suitable genetic transformation methods, and the discovery or establishment of handy model systems, such as *R*. *microsporus*, will help decipher the roles of these complex microbial symbioses and their evolution.

Once we have a deeper understanding of microbial endosymbionts in fungi, we will be able to design symbiotic systems that synergize the capabilities of fungi, bacteria, and viruses. These synthetic fungal holobionts could help us improve our crops to face climate change; restore eroded or contaminated soils; produce high-valued chemicals, enzymes, and other biomolecules and biomaterials; and increase the recycling of organic matter and plastics.
